# Decreased Blood Glucose and Lactate: Is a Useful Indicator of Recovery Ability in Athletes?

**DOI:** 10.3390/ijerph17155470

**Published:** 2020-07-29

**Authors:** Woo-Hwi Yang, Hyuntae Park, Marijke Grau, Oliver Heine

**Affiliations:** 1 Graduate School of Sports Medicine, CHA University, Seongnam-si, Gyeonggi-do 13503, Korea; 2 Department of Health Care and Science, College of Health Science, Dong-A University, Busan 49315, Korea; 3 Department of Molecular and Cellular Sports Medicine, Institute of Cardiovascular Research and Sports Medicine, German Sport University Cologne, 50933 Cologne, Germany; m.grau@dshs-koeln.de; 4 Olympic Training Centre Rhineland, 50933 Cologne, Germany; heine@osp-rheinland.de

**Keywords:** aspartate transaminase, Cori cycle, hepatic blood flow, oxaloacetate, phosphoenolpyruvate carboxykinase, pyruvate dehydrogenase

## Abstract

During low-intensity exercise stages of the lactate threshold test, blood lactate concentrations gradually diminish due to the predominant utilization of total fat oxidation. However, it is unclear why blood glucose is also reduced in well-trained athletes who also exhibit decreased lactate concentrations. This review focuses on decreased glucose and lactate concentrations at low-exercise intensity performed in well-trained athletes. During low-intensity exercise, the accrued resting lactate may predominantly be transported via blood from the muscle cell to the liver/kidney. Accordingly, there is increased hepatic blood flow with relatively more hepatic glucose output than skeletal muscle glucose output. Hepatic lactate uptake and lactate output of skeletal muscle during recovery time remained similar which may support a predominant Cori cycle (re-synthesis). However, this pathway may be insufficient to produce the necessary glucose level because of the low concentration of lactate and the large energy source from fat. Furthermore, fatty acid oxidation activates key enzymes and hormonal responses of gluconeogenesis while glycolysis-related enzymes such as pyruvate dehydrogenase are allosterically inhibited. Decreased blood lactate and glucose in low-intensity exercise stages may be an indicator of recovery ability in well-trained athletes. Athletes of intermittent sports may need this recovery ability to successfully perform during competition.

## 1. Introduction

Clinical physicians and sports scientists have used lactate threshold (LT) tests for over fifty years because their application is considered extremely useful for recommendations on individual exercise intensity in cardiac patients and trained athletes [1,2]. Endurance athletes regularly undergo these tests in order to control individual exercise intensity during endurance training [1,3,4,5,6]. Both respiratory and metabolic parameters are commonly utilized to identify the anaerobic threshold [1] and oxygen uptake (VO_2_) during exercise performance influenced by the percentage of maximal oxygen uptake (VO_2max_) at LT. The workout test is performed either on a bicycle ergometer or on a treadmill applying different steps [1,7].

The ramp test is applied to determine VO_2max_ and lactate values at each step in order to analyze the metabolic system and physiological performance [8]. The number of scientific studies on LT has increased enormously and among the diagnostics of endurance performance in sports, submaximal exercise is probably one of the most relevant [1,2,5,9,10,11,12,13]. For instance, increased exercise intensity at four millimoles per liter lactate was commonly observed as the lactate threshold in endurance-trained athletes, and this value is highly associated with the potential maximal lactate steady state level (MLSS) [4,14,15]. A rightward shift of the exponential lactate curve can generally be interpreted as improved endurance capacity [2,16,17,18]. Furthermore, validated LT concepts such as aerobic-anaerobic transition using lactate and gas exchange parameters were applied and refined by several scientists [2,4,10,12,18,19,20,21,22,23,24].

To measure the exercise capacity, numerous studies have been focused on altered blood glucose concentrations following moderate-to-high-intensity exercise in LT tests. The metabolic changes in blood glucose concentration during low-intensity exercise in LT test are not analyzed [2,4,14,15,18,19,20,21,22,23,25,26,27,28,29,30,31,32,33]. Glucose 6-phosphate, supplied through breakdown of muscle glycogen and blood glucose, is metabolized to lactate and re-synthesized to adenosine triphosphate (ATP) by substrate-level phosphorylation reactions [34]. The blood glucose of endurance-trained athletes is decreased during the early stages of LT testing while blood lactate concentration (below lactate baseline concentration; LTAer or < two millimoles per liter) is also reduced. This exercise area is commonly referred to as regenerative endurance training [2]. In these low exercise stages, it seems likely that blood lactate concentrations gradually decrease as a result of the predominance of total fat oxidation [2,14,25,26,27]. In terms of energy metabolism, fat is also used as an energy source and represents the main energy source in moderate exercise under aerobic conditions. However, fat oxidation cannot predominantly be used to meet the energy demand during high-intensity exercise. Under this condition, carbohydrate oxidation represents the primary source of energy [25,35]. In turn, at low-intensity, triglycerides in adipocytes are hydrolyzed into glycerol and free fatty acids (lipolysis) which are then converted into acetyl-CoA by ß-oxidation in the mitochondria. At low-intensity exercise levels of 25% VO_2max_, plasma fatty acids are delivered for energy production [25,36]. In light of this, it is understandable why lactate values in blood begin to decrease at this exercise intensity as more pyruvate and lactate are used aerobically than are generated via anaerobic glycolysis [14]. However, the reduction in blood glucose during low-intensity exercise is difficult to explain. Blood glucose concentrations are usually increased incrementally with exercise from low to high intensity because carbohydrate metabolism partly contributes to aerobic glycolysis during low-intensity exercise [14,25].

The aim of this literature review was to describe possible relationships between exercise intensity, glucose and lactate at the low-intensity exercise stages of the LT test. To date, it is unclear why blood glucose is reduced while lactate values are also decreased during low-intensity exercise. Therefore, comprehensive aspects of the underlying physiological and molecular biologic background are considered. We suggest that decreased blood glucose and lactate at low-intensity exercise (LT test) are relevant signals for the recovery ability of well-trained athletes in intermittent and endurance sports.

## 2. Materials and Methods

Literature studies were performed using online data bases including Scopus, PubMed (Medline) and Web of Science and published articles were retrieved (1929–2019).

Major keywords regarding lactate threshold test (“LT”, “lactate threshold”, “MLSS”, “endurance”, “aerobic”, “anaerobic” and “recovery”) and physiological and biochemical reactions occurring during low-intensity exercise (“glycolysis”, “gluconeogenesis”, “glycogenesis”, “lactate metabolism”, “glucose metabolism”, “MCT”, “fat oxidation”, “oxaloacetate”, “pyruvate”, “AMPK”, “hepatic blood flow”, “skeletal muscle blood flow”, “skeletal muscle lactate output” and “hepatic lactate uptake”) were used in diverse combinations. Original full-text articles and reviews in English language published in scientific journals were included. Articles describing human and animal species were included. Conference articles, posters and studies with information overlapping with another publication were excluded. Based on a review of overlapping articles, the most recent or the most comprehensive articles were selected.

**Figure 1:**
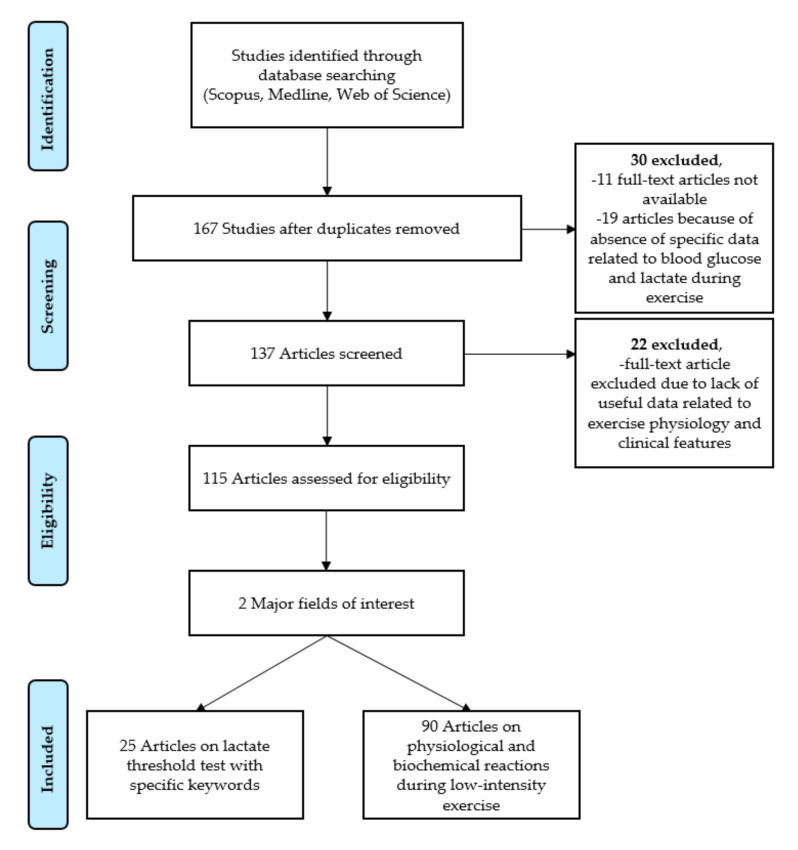
Flow chart outlining the literature search strategy.

After the initial searches identified articles, of which 167 were screened from the aforementioned databases. 30 articles were excluded because of unavailable full-text articles (11) and absence of specific data related to blood glucose and lactate without exercise (19). Of these, 115 articles were screened for eligibility, while 22 were excluded due to lack of useful data related to exercise physiology and clinical features (Figure 1). One author (W.-H.Y.) reviewed the titles and abstracts of studies and the remaining 167 articles using the foregoing search strategy. Another author (H.P.) reviewed the article inclusion/exclusion criteria. Eligible articles were retrieved and independently assessed by two authors (W.-H.Y. and H.P.). The disagreement between authors over the eligibility of remaining articles was resolved through discussion with other collaborating authors (M.G. and O.H.). Furthermore, two authors (W.-H.Y. and H.P.) independently extracted data from articles based on study features and populations, type of intervention, measurement procedure and outcomes.

## 3. Utilization of Fat Oxidation During Low-Intensity Exercise

The entire energy system, including phosphagens, glycolysis and oxidative phosphorylation, is simultaneously used during all levels of exercise intensity. In general, it seems important which energy system is predominantly used during different exercise intensities and exercise volumes. The energy storage of human fat is effectively unlimited during exercise [37]. Accordingly, one gram of fat provides about 40.79 kJ of energy. Very lean individuals of 70 kg and 10% body fat approximately have 285.56 kJ of endogenous fat energy [38]. With regard to low-intensity exercise, the oxidative metabolism from carbohydrate and fat is predominant. Adipocytes store large amounts of energy in the form of triglycerides which amount to 200–625 Megajoule (MJ) in humans with normal body compositions of 10–30% body fat [25,36]. The energy expenditure derived from fat comes from various sources including plasma fatty acids from lipolysis in adipose tissue, fatty acids liberated from hydrolysis of circulating very low density lipoprotein (VLDL)-triacylglycerol and fatty acids from lipolysis of triacylglycerol located in lipid droplets in the skeletal muscle [39]. Plasma triglycerides are used as a crucial energy source in the muscle. However, when triglyceride in muscle cells are catalyzed by lipoprotein lipase, their contribution to energy demands during high-intensity exercise is limited [40].

During low-intensity exercise (25% VO_2max_), overall energy is obtained from plasma fatty acids with an additional small contribution from blood glucose. The rate of plasma fatty acid oxidation is similar to the rate of fatty acid oxidation (26 μmol·kg**^−^**^1^·min**^−^**^1^) in endurance-trained athletes. Furthermore, an increase in exercise intensity from 25% to 85% VO_2max_ resulted in a progressive decline of fatty acid oxidation along with a proportional reduction of its concentration in blood [25]. This was due to insufficient transport of outflowing blood and albumin from adipose tissue into the systemic circulation [36,41].

## 4. Lactate, Glucose, Enzymatic Responses and Cori Cycle During Exercise

Lactate is produced during glycolysis, which is one of the metabolic pathways through which glucose can be utilized to provide energy. Lactate production from glycolysis occurs in muscle when exercise intensity increased [27]. Anaerobic conditions were not essential for the production of lactate in animal experiments (tail shaker muscle; western diamondback rattlesnake) [42] thus indicating that energy systems (phosphagen, glycolytic and oxidative) started to work simultaneously while the dissociation between lactate and hypoxic or anoxic conditions was orderly conformed [27]. Another study using the same model in ischemic and normoxic situations showed that increased rates of glycolysis could occur independently of O_2_ [43]. Such muscle conditions indicated the capability for exercise without fatigue [27] because of high blood flow rates that allowed the rapid turnover of H^+^ and lactate within the cell (and also other metabolites that may be involved in the fatigue process) [27,44]. These results indicated that, in addition to lactate production during anoxic or hypoxic situations, lactate was also produced as a metabolite due to adequate oxygenation [27].

Formerly, the understanding of lactate physiology was that lactate transport took place through simple diffusion (e.g., in the bloodstream) from cellular compartments to the blood. Increased lactate concentrations were believed to be a consequence of glycolytic flux rates [45,46,47,48]. In addition, previous studies had shown that three pathways were involved in lactate transport in red blood cells (RBC)—(i) H^+^ coupled transporter, (ii) band 3 protein Cl**^−^**/HCO_3_^-^ mediated exchange with inorganic anions and (iii) passive diffusion of lactate across the lipid bilayer [49,50,51].

Nowadays, monocarboxylate transport (MCT) proteins (14 isoforms in total) are known to play critical roles in lactate transport. Cluster of differentiation 147 (CD147) functions as an ancillary protein that chaperones MCT1 and MCT4 to the cell membrane (muscle, red blood cell and liver). Human, rat and horse muscles express MCTl and MCT4. Both MCT1 and MCT4 need of an ancillary protein CD147 for their activity [52,53,54]. MCT1 and 4 are the predominant MCT transporters in human skeletal muscle while MCT2 is prominently expressed in the liver and brain [55,56]. MCT1 is coordinately expressed with isoforms of lactate dehydrogenase (LDH). High levels of MCT1 and LDH are found in oxidative muscle fibers [57]. In addition, MCT1 is the most important protein for lactate transport into or out of RBC [58,59]. In contrast, the low affinity transporter MCT4 was shown to be relevant for the net export of lactate from the cell which was predominantly expressed in glycolytic type IIA fibers [60]. MCTs transfer lactate into and out of cells and other organs such as liver, kidney, heart and brain [61,62]. These are now known as lactate shuttle mechanisms. The intracellular lactate shuttle mechanism is based on mitochondria-localized LDH (mLDH) for the re-synthesis between lactate and pyruvate [63]. During lactate production at rest and during submaximal exercise, pyruvate is converted to lactate by lactate dehydrogenase (LDH and mLDH) reaction. In addition, lactate can be reversibly converted to pyruvate by the intracellular lactate shuttle mechanisms [27,61,64].

The liver is capable of eliminating lactate during exercise [65,66]. The Cori cycle, refers to the metabolic pathway of lactate-produced by anaerobic glycolysis in the muscle cells-moved to the liver and converted to glucose in order to ultimately return to the muscles [67]. Intensive exercise may impair the Cori cycle resulting in increased blood lactate concentrations which can be affected by decreased hepatosplanchnic blood flow [68]. Nielsen et al. [69] reported that arterial lactate was decreased because of reductions in lactate release from the working muscles during prolonged exercise (2 h and ~70% of VO_2max_, respectively). In contrast, liver clearance of lactate was maintained during a 2 h exercise phase. Lactate release by legs was significantly increased with increased work rate (~90% of VO_2max_ during 20 min). However, the uptake of hepatic lactate constituted only one-tenth of the leg lactate production compared with 25% during prolonged exercise, while hepatic blood flow was markedly decreased, and leg blood flow increased. This reduction in hepatic extraction ratio may influence the rise in arterial lactate concentrations when exercise intensity is increased. On the other hand, leg lactate output and hepatic lactate uptake were similar (0.5 ± 0.3 and 0.55 ± 0.25 mmol·min*^−^*^1^, respectively) and the hepatic blood flow was accordingly increased during a recovery period (20 min) between exercises [69]. This study result showed that a two-third reduction in hepatic blood flow was among the most distinct changes during high-intensity exercise. With more intensive sympathetic activation and a cardiac output of more than 30 L·min*^−^*^1^, indocyanine green dye (ICG) eliminations may even approximate zero [70]. Therefore, a reciprocal relationship existed between liver and leg blood flow. During resting condition, hepatic blood flow was 19% of cardiac output which decreased to 2% during high-intensity exercise. This indicates that splanchnic organs contribute as a “blood donor” to the systemic circulation [69,71].

Glucose utilization and total glucose production are balanced by the concentration of glucose in arterial blood. As described above, the Cori cycle is responsible for lactate to glucose conversion in the liver [67]. However, if the hepatosplanchnic blood flow reaches a minimum, resulting in a reduction in hepatic venous O_2_ saturation to 6%, the contribution of the Cori cycle to glucose production appears to decrease during exercise [68]. During prolonged exercise, relative hypoglycemia may emerge although the rate of glucose appearance is significantly increased [72,73,74,75]. Therefore, muscle glucose uptake can be increased with time during prolonged exercise [65,69]. During high-intensity exercise, leg glucose uptake was increased while hepatic glucose output was significantly decreased (6.2 ± 1.3 and 1.9 ± 0.41 mmol·min*^−^*^1^, respectively). Furthermore, another study outcome showed that when exercise intensity was higher than 50% of VO_2max_ the rate of gluconeogenesis was decreased because of the reduced hepatic blood flow [45]. In comparison to these levels, leg glucose uptake was markedly lower than hepatic glucose output during rest and recovery times (0.3 ± 0.1, 1.9 ± 0.5 and 1.55 ± 0.23, 2.34 ± 0.75 mmol·min*^−^*^1^, respectively) [69]. During recovery, despite hepatic blood flow being relatively increased, the Cori cycle (gluconeogenesis) may be insufficient to provide the needed glucose for maintaining blood glucose concentrations.

The mechanism of attenuation of gluconeogenesis by sympathetic nervous system and upregulation of glycogenolysis still remains unclear [76,77]. The hepatic artery is sustained with α- and β-receptors [78,79]. A high level of epinephrine could cause an increase in hepatic glucose production, partly owing to an increased supply of gluconeogenic substrates such as alanine—and partly associated with a direct action on the liver cells [80]. In contrast, exercise with β-receptor blockade led to decreased hepatic uptake of gluconeogenic precursors, decreased lactate uptake and increased glucose output [76]. Furthermore, interleukins were released from active muscle during exercise and these are relevant for hepatic glucose production [77].

Decreased hepatosplanchnic blood flow may reduce the available number of hepatic sinusoids. Norepinephrine decreases the hepatic blood volume—even the plasma volume in hepatic sinusoids may be influenced [81]. Blood flow reductions of 30–40% during hemorrhage in the pig resulted in a reduction of hepatic norepinephrine uptake which induced a partial sinusoidal collapse [82]. In addition, Nielsen et al. [68] showed that a decreased intrinsic hepatic elimination of ICG during exercise caused a reduction of active sinusoidal area in human.

According to the aspects described above, lactate and glucose concentrations in well-trained endurance athletes gradually decreases during low-intensity exercise. As a large part of gluconeogenesis, accumulating lactate may be predominantly oxidized during rest or low-intensity exercise in the liver. However, this may be insufficient to produce appropriate glucose concentrations because of the low concentration of lactate and in addition the large energy source derived from fat between rest and low-intensity exercise. Further assumptions and available evidence are discussed in upcoming sections.

## 5. Allosteric Regulation between Glycolysis and Gluconeogenesis

Allosteric regulation between glycolysis and gluconeogenesis can depend on the release of insulin, glucagon and cortisol. The role of glucagon and cortisol is to increase the concentration of blood glucose, while in opposition, insulin decreases blood glucose [83,84,85].

Hormonal regulation of metabolic reactions in the liver occurs by two major mechanisms. First, glucagon and β-adrenergic agonists interact with plasma membrane receptors which are associated with adenylate cyclase. The activity of these membrane-bound receptors increases intracellular cyclic adenosine monophosphate (cAMP) which drives the activation of cAMP-dependent protein kinase and catalyzes the phosphorylation of many protein substrates. Finally, these cascading events induce the activation of gluconeogenesis and inhibit glycolysis [86,87].

Second, those hormones act via alterations in intracellular calcium ion (Ca^2+^) concentration levels. Alpha-adrenergic agonists, vasopressin and angiotensin interact with their specific plasma membrane receptors to induce two intracellular messengers, myoinositol-1,4,5-trisphosphate and 1,2-diacylglycerol [88]. These increase intracellular Ca^2+^, which in combination with calmodulin or other effectors, stimulates a number of Ca2^+^-associated protein kinases including Ca^2+^/calmodulin-dependent protein kinase, phosphorylase kinase and protein kinase C. Furthermore, protein kinase catalyzes phosphorylation of many protein substrates which lead to alterations in gluconeogenic and glycolytic flux [86].

Phosphoenolpyruvate is partly recycled to pyruvate (for short-term hormonal regulation), during gluconeogenesis in perfused liver and isolated hepatocytes [89,90,91,92]. This flux is strongly inhibited by glucagon and cAMP. Liver-pyruvate kinase (PK), an allosteric enzyme, inhibits sigmoidal kinetics with regard to phosphoenolpyruvate (PEP). This enzyme is allosterically activated by fructose 1, 6-bisphosphate (Fru-1, 6-P2) and repressed by alanine and ATP. The in vitro studies of physiological concentrations of alanine, ATP and PEP, showed that these enzymes would be inhibited if they are not activated by Fru-1, 6-P2 [93,94].

From rest through moderate intensity exercise, ATP is primarily generated from fat oxidation [27]. The increased plasma/blood glucose concentration inhibits non-esterified fatty acid (NEFA) released by adipose tissue, by secreting insulin. In turn, elevated NEFA can decrease insulin and glucose concentrations and fatty acids are predominantly released and oxidized [95]. Moreover, Khani et al. [84] suggested that the infusion of cortisol increased NEFA. Therefore, it is important to recognize that cortisol increases lipolysis and NEFA concentrations. They also found moderate correlations between NEFA and gluconeogenesis in different observed groups (r = 0.599–0.665). These results do not indicate cause and influence. However, associations between NEFA and the rate of gluconeogenesis suggested existence of a relationship [96,97,98]. Mitochondrial acetyl-CoA acts as a key allosteric activator of pyruvate carboxylase (PC) which is activated from increased fatty acid. This allosteric activator leads to increased production of oxaloacetate for gluconeogenesis oxidation [99].

## 6. Regulation of AMPK in Energy Metabolism

AMP-activated protein kinase (AMPK) is a key regulator of physiological energy dynamics and functions by limiting anabolic, while facilitating catabolic pathways. AMPK is a heterotrimer and possesses an α (α_1_ and α_2_)-catalytic subunit and β (β_1_ and β_2_) and γ (γ_1_ and γ_2_ and γ_3_)-catalytic subunits. Three subunit combinations exist in human skeletal muscle. These are α_1_/β_2_/γ_1_, α_2_/β_2_/γ_1_ and α_2_/β_2_/γ_3_ [100]. γ_3_ is expressed predominantly in glycolytic skeletal muscle while there is very low level of γ_3_ expression in oxidative muscles. The role of α_1_, α_2_ and γ_3_, during contraction of skeletal muscle or while exercising, in glucose metabolism has been broadly investigated [100,101].

The function of AMPK is to be a sensor in most tissues and organs including liver, skeletal muscle, heart, hypothalamus and adipose tissue. AMPK works by influencing enzymatic activities directly and is involved in biosynthesis of carbohydrates, lipids and proteins. AMPK regulates glucose and lipid metabolism and activates hepatic AMPK causing increased fatty acid oxidation [102]. AMPK is activated by a variety of exercise stresses. These typically alter cellular AMP: ATP ratio, either by increasing ATP or decreasing ATP production due to hypoxia, glucose deprivation or inhibition of mitochondrial oxidative phosphorylation [103]. There are conflicting results related to fatty acid oxidation induced by exercise. The concentration of AMPK induces fatty acid oxidation by utilizing an AMPK activator, 5-aminoimidazole-4-carboxamide-1-β-D-ribofuranoside (AICAR). In common with exercise, in skeletal muscles, AICAR affects the phosphorylation of acetyl-CoA carboxylase 2 (ACC2) which is an isoform of squamous cell carcinoma (SCC). AICAR increases the rate of uptake of long-chain fatty acids in cardiac myocytes via translocation of fatty acid translocase (FAT)/CD36 to the sarcolemma [104]. This mechanism reduces the level of malonyl-CoA and releases the inhibition of fatty acids uptake into mitochondria via carnitine palmitoyl transferase 1, resulting in fatty acid oxidation [101,105].

However, α_2_-AMPK activation is unnecessary for increasing fatty acid oxidation during exercise of low-intensity [101]. Miura et al. [101] suggested that in skeletal muscle, α_2_-AMPK may not have a major role in the shift to fatty acid oxidation from glucose oxidation while fasting, because the respiratory quotient ratio and utilization of oxygen during the fasting state remained constant between α_1_-AMPK -dominant-negative transgenic mice and wild-type littermates. Peripheral lipolysis can be maximally stimulated at the lowest exercise intensity in humans as well as at 25% of VO_2max_, whereas uptake of plasma glucose and oxidation of muscle glycogen increases with exercise intensity [106]. At 30% of VO_2max_, such as during prolonged low-intensity exercise, free fatty acid oxidation increases progressively while glucose oxidation is decreased [107]. Nevertheless, the increased activity of α_2_-AMPK is not necessary for increases in oxidation of fatty acid in skeletal muscle during endurance performance [101].

## 7. Fat Oxidation Stimulates Gluconeogenesis and Can Decrease Glucose in Blood

Low-intensity exercise causes fat oxidation that increases gluconeogenesis which occurs mostly in the liver and kidney [25,36,108,109,110,111,112,113]. In both, the liver and kidney, glycerol can be converted directly to glycerol 3-phosphate by glycerol kinase when glycerol is plentiful. Glycerol 3-phosphate is further converted to dihydroxyacetone phosphate by glycerol 3-phosphate dehydrogenase for gluconeogenesis. The direct conversion to glycerol 3-phosphate from free glycerol is believed to be trivial in skeletal muscle as well as in adipose tissue due to lower activity of glycerol kinase [114,115,116,117]. However, Guo et al. [118] suggested that the capacity for using blood glycerol for intracellular triacylglycerol (TG) synthesis in skeletal muscle is greater than was seen in previous studies [114,115,116,117]. Indeed, glucose was a constitutive substrate for muscle TG glycerol synthesis which may have provided TG derived glycerol with carbons [118]. The almost complete loss of ^3^H label in relation to 14C from blood glucose in muscle TG glycerol suggests (calculation of ^3^H label from glucose) that glucose passed through PC catabolized reactions and thus gluconeogenic precursors may also have paved their way to triglyceride glycerol [118,119]. Consequently, a pattern of preference to blood glycerol via blood glucose for TG glycerol synthesis in type 1 fiber-rich muscle was observed and its glycerol kinase activity was higher than in other types of muscle. Blood glycerol for intramuscular synthesis of TG glycerol is associated with the capacity of muscle to oxidize as well as store fatty acids [115,118].

Fatty acid availability is increased by mobilization of triglycerides in liver and adipose tissue. Increased fatty acid oxidation induces an increased rate of ketone-body formation and increased tissue concentrations of acetyl-Coenzyme A (CoA), fatty acyl-CoA and reduced NAD^+^ [108]. The oxidation of pyruvate is decreased due to inhibition of pyruvate oxidase by acetyl-CoA or competitive CoA between pyruvate oxidase and the fatty acid oxidation system [120,121]. Accordingly, the complex mechanisms of pyruvate dehydrogenase include inhibition of end products by increases in concentration of mitochondrial acetyl-CoA, NADH and ATP. These can originate from fatty acid oxidation as well [122,123]. Pyruvate is converted to acetyl-CoA by pyruvate dehydrogenase (PDH). However, increased mitochondrial acetyl-CoA from fatty acid ß-oxidation activates pyruvate dehydrogenase kinases (PDHKs 1–4) and PC, which results in inhibition of PDH [108,124]. This enables the entrance of acetyl-CoA, derived from fatty acid oxidation in the first span of the tricarboxylic acid (TCA) cycle, to generate citrate. However, employment of the second span of the TCA cycle becomes reliant on NADH and FADH_2_ re-oxidation originating from fatty acid ß-oxidation [125].

Fatty acid oxidation can involve increased acetyl-CoA and inhibition of PDH. The TCA cycle must be substituted to allow continued function (anaplerosis) if its anions are removed. Due to inhibited PDH, PC is the major anaplerotic enzyme which immediately synthesizes oxaloacetate from pyruvate in the mitochondria [126]. During stimulated gluconeogenesis, the oxaloacetate concentration can fall due to increased PC activity. This can be explained by conversion of pyruvate into oxaloacetate that is still formed to malate. Thus, the metabolic production of the pyruvate carboxylase reaction may be related to the sum of malate and oxaloacetate [108,127,128]. In many tissues, the activity of PC is high (e.g., 10 to 12 units per gram in liver) and acetyl-CoA plays a role as a positive allosteric regulator of the enzyme, respectively [126].

This anaplerotic mechanism is mandatory during gluconeogenesis and lipogenesis when the reversible reaction from oxaloacetate to malate in the cytosol takes place with the aid of the malate–aspartate shuttle for gluconeogenesis or citrate for lipogenesis (oxaloacetate to cytosol, acetyl-CoA, malonyl-CoA) which exists in mitochondria and is still metabolized from glucose or fatty acids. Therefore, malate, but not oxaloacetate can traverse the inner membrane of mitochondria. Additionally, formation of malate is promoted by increased delivery of NADH from fatty acid oxidation [129].

Aspartate is converted to oxaloacetate to recruit cytosolic oxaloacetate by cytosolic aspartate aminotransferase. Hence, the effect of net redox-reaction of malate–aspartate shuttle is the oxidation of NADH to NAD^+^ in cytosol and reduction to NADH in the matrix. Accordingly, malate, aspartate and citrate are transferred precursors for oxaloacetate to gluconeogenesis [125,126].

It is equally relevant to remove TCA cycle intermediates and to avoid accumulated anions in the mitochondrial matrix. Cataplerotic reactions relate to disposal of TCA cycle intermediates. Phosphoenolpyruvate carboxykinase (PEPCK), which highly important in cataplerosis, generates PEP from oxaloacetate to be utilized for gluconeogenesis in the liver and kidney. Pyruvate is transported into the mitochondria where it is then converted into oxaloacetate or acetyl-CoA, respectively by PC and PDH. Mitochondrial oxaloacetate depends largely upon the distribution of PEPCK between cytosol and mitochondria [130]. The increase in phosphoenolpyruvate concentration is associated with decreased oxaloacetate concentration which may indicate activation of PEPCK [108,131]. The PEP from glycolysis otherwise can be converted to pyruvate that is decarboxylated to acetyl-CoA for ensuing oxidation to carbon dioxide (CO_2_) in the TCA cycle of muscle [126,132].

From muscle, glutamine can be transported to the kidney where ammonia is formed by utilization of the amino and amide groups. For generation of ammonia, glutamine goes through anaplerotic reactions to build α-ketoglutarate which joins the TCA cycle and is consequently metabolized to malate. Malate is further oxidized in the cytosol to oxaloacetate and to PEP and then to glucose [126]. The gluconeogenic pathway in liver and kidney is as follows: PEP, 2 phosphoglycerate, 3 phosphoglycerate, 1.3-bisphosphoglycerate, glyceraldehyde 3-phosphate (G3P) ↔ dihydroxyacetone phosphate (DHAP), fructose 1.6-bisphosphate, fructose 6-phosphate, glucose 6-phosphate and glucose [126].

**Figure 2:**
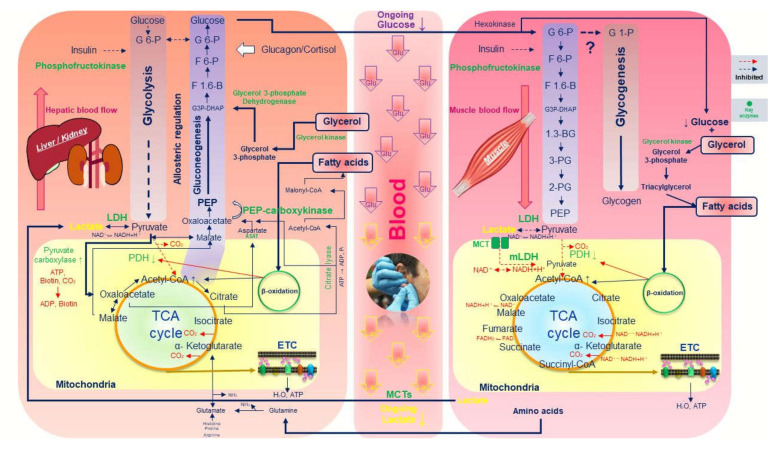
Summarized illustration of all described factors for decreased blood glucose and lactate values during the initial stages of lactate threshold test. ADP—adenosine diphosphate; ATP—adenosine triphosphate; ASAT—aspartate transaminase; DHAP—dihydroxyacetone phosphate; ETC;—electron transport chain; FAD—flavin adenine dinucleotide; F 1;6-B—fructose 1;6-bisphosphate; F 6-P—fructose 6-phosphate; G3P—glyceraldehyde 3-phasphate; G 6-P—glucose 6-phosphate; LDH—lactate dehydrogenase; PDH—pyruvate dehydrogenase; PEP—phosphoenolpyruvate; P_i_—inorganic phosphate; MCT—monocarboxylate transporter; NAD—nicotinamide adenine dinucleotide; 1;3-BG—1;3-bisphosphoglycerate; 2-PG—2 Phosphoglycerate 3-PG—3 phosphoglycerate.

Subsequently, reduced blood glucose concentration at the initial stages of low-intensity exercise (LT test) may occur because the glucose, through gluconeogenesis, seems to be transported to muscle cells via blood as an ongoing process and may be used as substrate for muscle TG glycerol synthesis. However, it is unclear whether this transferred blood glucose enters glycolysis or glycogenesis in muscles. The enzyme activity in human is similar to animals like the rat. Earlier studies were conducted by investigations using isotope tracers and arterial-venous difference. Although outstanding studies of gluconeogenesis were developed, phenomena in humans were never investigated because of technical limitations such as gluconeogenesis from lactate [64]. The factors mentioned above are summarized in Figure 2.

## 8. Conclusions

It is already known that fat oxidation is predominantly utilized to perform low-intensity exercise. This exercise area is crucial for estimating the recovery ability of athletes. During the low-intensity exercise, the accrued resting lactate may predominantly be transported via blood from muscle cells to the liver/kidney (ongoing moment) while lactate from muscle cells is less oxidized by the intracellular lactate shuttle mechanism [45,64]. Furthermore, increased hepatic blood flow according to relatively more hepatic glucose output than glucose output of skeletal muscle and similar remained hepatic lactate uptake and lactate output of skeletal muscle during recovery time may support aspects of the predominant activation of gluconeogenesis (Cori cycle). However, it may be insufficient to induce the production of needed glucose because of the low concentration of lactate and the large energy source from fat between rest and low-intensity exercise. Insufficient sympathetic drive also may influence blood glucose and lactate concentrations [68,69,133,134].

Fatty acid oxidation activates key enzymes and hormonal responses of gluconeogenesis such as PK, PC, PEPCK, glucagon, cortisol and other associated regulators such as cAMP and intracellular Ca^+^, while glycolysis-related enzyme such as PDH are allosterically inhibited [93,94,99,108,126,127,128,130,131,132].

The efficient use of fat oxidation during low-intensity exercise and its effect during LT test exhibited a rightward shift of the exponential lactate curve. This can be interpreted as improved regenerative ability, lactate threshold and endurance capacity [2,16,17,18]. Hence, decreased blood lactate and glucose may be a signal of efficient utilization of fat oxidation and improved recovery during low-intensity exercise. In particular, athletes of intermittent sports may need this recovery ability to improve performance in competition. The efficiency of fat oxidation during low-intensity exercise in athletes may be important to improve the regeneration of exercise performance between and during competition after highly intensive exercise load. In addition, strength athletes such as weight lifter may need this recovery ability to optimize the repeated high intensity training session because of the need for ATP re-synthesis.

Athletes with a relatively poor endurance capability and the general population may show increased blood glucose concentrations during low-intensity exercise. It indicates that they also use significant amounts of glucose to perform low-intensity exercise. Athletes and the general population need to low-intensity exercises which activate the corresponding enzymes via fat oxidation resulting in enhanced endurance and recovery.

Studies and findings of above-mentioned review were actively investigated and this review can be a first step toward identifying the associations between exercise intensity, blood glucose and lactate at the low-intensity exercise stages of LT test. Further studies are expected to investigate how these key enzymes and hormonal responses during low-intensity exercise are actually activated in humans with regard to gluconeogenesis.

## Abbreviations

AICAR.	Aminoimidazole-4-carboxamide-1-β-D-ribofuranoside
ATP	Adenosine triphosphate
AMPK	Adenosine monophosphate-activated protein kinase
Ca^2+^	Calcium ions
cAMP	Cyclic adenosine monophosphate
FADH_2_	Flavin adenine dinucleotide
Fru-1,6-P_2_	Fructose 1,6-bisphosphate
ICG	Indocyanine green dye
LT	Lactate threshold
mLDH	Mitochondria-localized lactate dehydrogenase
MCT	Monocarboxylate transport
MLSS	Maximal lactate steady state
NADH	Nicotinamide adenine dinucleotide
NEFA	Non-esterified fatty acid
PDH	Pyruvate dehydrogenase
PC	Pyruvate carboxylase
PEP	Phosphoenolpyruvate
PEPCK	Phosphoenolpyruvate carboxykinase
PK	Pyruvate kinase
SCC	Squamous cell carcinoma
TCA	Tricarboxylic acid
TG	Triacylglycerol
VO_2_	Oxygen uptake
VO_2max_	Maximal oxygen uptake
